# SLC43A2-mediated immune cell infiltration: a potential therapeutic target in acute myeloid leukemia

**DOI:** 10.3389/fimmu.2025.1655766

**Published:** 2025-09-25

**Authors:** Mengnan Li, Tao Zhang, Bing Wang, Jile Liu, Mohan Zhao, Yanyu Jiang, Yu Zhang, Mingfeng Zhao

**Affiliations:** 1 School of Medicine, Nankai University, Tianjin, China; 2 Department of Hematology, Tianjin First Central Hospital, Affiliated Hospital of Nankai University, School of Medicine, Nankai University, Tianjin, China; 3 First Central Clinical College, Tianjin Medical University, Tianjin, China

**Keywords:** acute myeloid leukemia, TCGA, SLC43A2, T cells, immunity

## Abstract

**Background:**

Tumor cells compete with T cells for methionine by overexpressing SLC43A2 (solute carrier family 43 member 2), leading to T cell exhaustion. However, the correlation between SLC43A2 and immune infiltration or malignant features in acute myeloid leukemia (AML) has rarely been explored.

**Methods:**

We obtained gene expression data and clinical information from 173 AML patients in The Cancer Genome Atlas (TCGA) database. Statistical analyses were performed using R (v4.0.5). Differential expression, immune infiltration, functional enrichment, and survival analyses were conducted using online databases, including GEPIA, GSCA Lite, Kaplan–Meier Plotter, KEGG, and TIMER2. *In vitro*, we established SLC43A2-knockdown cell lines and validated SLC43A2 expression via PCR. Flow cytometry was employed to analyze cell viability, counts, apoptotic cell ratios, and immune cell infiltration.

**Results:**

SLC43A2 was highly expressed in AML and associated with poorer survival. Enrichment analysis revealed that SLC43A2 is involved in lymphocyte activation, leukocyte adhesion, and immune response regulatory signaling pathways. Immune infiltration results showed that SLC43A2 positively correlated with immune exhaustion markers (all p < 0.05) and negatively correlated with CD8+ T, NK, and B cell infiltration (all p < 0.05). We constructed an SPI1-hsa-miR-31-5p-SLC43A2 transcriptional network to support the role of SLC43A2. *In vitro* experiments have shown that SLC43A2 is negatively correlated with the infiltration level of immune cells and positively correlated with the expression levels of PDCD1 and CTLA4. Moreover, lower SLC43A2 expression was associated with enhanced T cell cytotoxicity.

**Conclusion:**

The SLC43A2 gene may serve as a diagnostic, prognostic, and potential immune-related biomarker for AML patients. Blocking SLC43A2-associated signaling pathways could provide novel insights into immunotherapy for AML.

## 1 Introduction

Acute Myeloid Leukemia (AML) is a malignant hematologic disease originating from myeloid hematopoietic stem cells, characterized by the rapid proliferation of abnormal leukemic cells in the bone marrow and peripheral blood, which frequently leads to impaired normal hematopoietic function (1). The pathogenesis of AML is complex, involving multiple genetic mutations, chromosomal abnormalities, and digenetic alterations (2). These factors collectively result in blocked cell differentiation and uncontrolled proliferation. In recent years, with the in-depth investigation of the immune microenvironment in AML, immunotherapy has emerged as a novel therapeutic strategy and is gradually becoming a new direction for AML treatment (3). Immunotherapy activates or enhances the body’s own immune system to recognize and attack leukemic cells, including immune checkpoint inhibitors (4), CAR-T cell therapy (5), and immune modulators. However, immunotherapy for AML still faces numerous challenges, such as complex immune escape mechanisms, the presence of an immunosuppressive microenvironment and treatment-related toxicities (6). Therefore, in-depth exploration of the immune microenvironment in AML, identification of biomarkers that can effectively predict immunotherapy responses, and development of novel immunotherapeutic strategies are of great significance for improving the prognosis of AML patients.

Cancer cells exhibit the “Warburg effect,” undergoing fundamental metabolic changes that reduce their dependence on aerobic glycolysis (7–9). Instead of directing pyruvate into the tricarboxylic acid cycle and oxidative phosphorylation, cancer cells convert pyruvate into lactate. This metabolic reprogramming enables cancer cells to generate 40% new biomass (10). To support continuous biomass accumulation and cell division, cancer cells need to acquire amino acids from the tumor microenvironment. Non-essential amino acids can be synthesized from glucose or other amino acids, while essential amino acids cannot be synthesized within the cell and must be obtained from external sources (11). Therefore, the availability of essential amino acids is a major limiting factor for cancer cell homeostasis and growth. Immune cells rely on solute carrier transporters to transport metabolites involved in gene regulation and signal transduction (12).The research community has been focusing on several amino acid transporters, including SLC43A2 (solute carrier family 43 member 2), as potential therapeutic targets for cancer. SLC43A2 is a promising candidate target because it plays a key role in the transport of many essential amino acids, including methionine, branched-chain amino acids, and aromatic amino acids. Multiple studies have demonstrated that SLC43A2 is associated with the occurrence and development of various tumors (13, 14). A study previously published in Nature reported that SLC43A2 in tumor cells can alter T cell methionine metabolism, leading to T cell exhaustion. Inhibition of SLC43A2 in tumor cells can restore methionine metabolism in effector T cells, rescue their function, and improve anti-tumor immune responses in preclinical models (15).

However, to the best of our knowledge, the relationship between SLC43A2 and the tumor immune microenvironment as well as prognosis in AML has not been reported. It remains unclear whether SLC43A2 plays a role in regulating immune cell infiltration. Therefore, in this study, we investigated the predictive value of SLC43A2 in AML and elucidated its relationship with immunity. Additionally, we confirmed the biological functions and signaling pathways associated with SLC43A2 through Gene Set Enrichment Analysis (GSEA). To better understand the immune relevance of SLC43A2, we evaluated the associations between SLC43A2 expression and immune infiltration, tumor microenvironment, and prognosis. This study is expected to promote the development of new therapies and provide effective clinical biomarkers for AML.

## 2 Materials and methods

### 2.1 Public databases

The Cancer Genome Atlas (TCGA) is a large-scale cancer genomics research project conducted in the United States, which has collected multi-omics data for over 30 types of tumors. First, we obtained gene expression data and detailed clinical information for 173 patients with AML from the TCGA official website (https://www.cancer.gov/tcga). The clinical information included age, sex, cytogenetic risk, NPM1 mutation status, and RAS mutation status. Second, we retrieved RNA sequencing data for 70 normal blood samples from the Genotype-Tissue Expression (GTEx) database (http://www.gtexportal.org). Additionally, we collected two datasets (GSE65409 and GSE97485) from the Gene Expression Omnibus (GEO) database (http://www.ncbi.nlm.nih.gov/geo). Data preprocessing was performed using the “Bioconductor” R package (version 3.10).The differential expression analysis of the target gene SLC43A2 was analyzed and visualized by the “limma”, “ggplot2” and “ggpubr” package.

### 2.2 Gene expression analysis

We further validated the expression differences of SLC43A2 between AML tumor samples (n = 173) and control blood samples (n = 70). Public microarray data for AML and normal controls were obtained from TCGA database and the GEO database (GSE65409 and GSE97485). Differential expression of SLC43A2 mRNA between AML patients and normal controls was analyzed using the “Bioconductor” R package and GEO2R. Statistical significance was determined by a P<0.05.

### 2.3 Survival analysis

To evaluate the prognostic significance of SLC43A2 in AML, we performed univariate survival analysis incorporating clinical variables such as patient age, sex, white blood cell count, cytogenetic risk, FLT3 mutation, NPM1 mutation, RAS mutation, and SLC43A2 mRNA expression levels. The correlation between SLC43A2 expression levels and mortality in AML patients was analyzed. Subsequently, Survival package and survminer package were used to estimate the overall survival between high and low expression levels of SLC43A2 expression. Through the “survival ROC” package in R, we evaluated the prognostic ability by AUC and ROC analysis.

### 2.4 LinkedOmics database analysis and enrichment analysis

The LinkedOmics database (http://www.linkedomics.org/login.php) is a multi-omics database that includes data from 32 cancer types and their clinical information. This database was selected to identify differentially expressed genes (DEGs) between AML and normal tissues in TCGA data. The constructed co-expression network was visualized using a volcano plot. A heatmap was used to display genes that were significantly positively or negatively correlated with SLC43A2 expression and their prognostic values. AML patients were divided into high/low expression groups based on the median level of SLC43A2 expression. DEGs were screened using thresholds of |log2 FC| > 1 and p-adjusted < 0.05. To elucidate the functional characteristics of these DEGs, Gene Ontology (GO), Kyoto Encyclopedia of Genes and Genomes (KEGG) (16, 17), and GSEA were performed to analyze the potential signaling pathways involved in these DEGs. GO, KEGG and GSEA enrichment analysis were conducted and visualized by R language programs “clusterProfler”,”org.Hs.eg.db”, “enrichplot”, “ggplot2”, “circlize”, “RColorBrewer”, “dplyr”, and “ComplexHeatmap” with 0.05 as P value filter. P < 0.05 was defined as statistically significant difference.

### 2.5 Transcription factor-miRNA-mRNA regulatory network

Transcription factors (TFs) targeting SLC43A2 were predicted based on the databases CHEA (https://maayanlab.cloud/Harmonizome/) and ENCODE (https://www.encodeproject.org/), as well as ChIP_Atlas (https://chip-atlas.org/) and GTRD (http://gtrd.biouml.org/). Binding sites for these TFs were predicted using JASPAR (https://jaspar.genereg.net/). MicroRNAs (miRNAs) targeting SLC43A2 were predicted using three distinct databases: miRWalk (RRID: SCR_016509; http://mirwalk.umm.uni-heidelberg.de/), TargetScan (https://www.targetscan.org/vert_80/), and mirDIP (RRID: SCR_016770; http://ophid.utoronto.ca/mirDIP/). The overlapping results from these predictions were visualized using the VennDiagram package (RRID: SCR_002414). Subsequently, the complementary sequences of the identified miRNAs were retrieved from miRDB.

### 2.6 Immune-related analysis

First, we utilized the ssGSEA algorithm from the “GSVA” R package (version 1.40.1) to calculate the infiltration levels of 24 immune cells in AML based on previously reported immune cell biomarkers (18). Subsequently, Spearman correlation analysis was performed to evaluate the correlation between SLC43A2 expression and the tumor infiltration levels in AML. We also assessed the associations between SLC43A2 and immune checkpoints in AML using Spearman correlation analysis. P< 0.05 was considered statistically significant.

### 2.7 SLC43A2 interaction network analysis

We constructed the protein-protein interaction (PPI) network of SLC43A2 using the STRING database (https://cn.string-db.org/). Additionally, we developed a gene network for SLC43A2 and predicted potential target genes using the GeneMANIA database (http://genemania.org/).

### 2.8 Cell experiments

#### 2.8.1 Cell culture

The human U937 cell line was obtained from the Culture Collection of the Chinese Academy of Sciences. T cells and peripheral blood mononuclear cells were isolated from patients diagnosed with AML. This study was approved by the Institutional Review Board of Tianjin First Central Hospital. The cell line and primary T cells were cultured in RPMI-1640 medium (Gibco, USA) supplemented with 10% fetal bovine serum (FBS; Biological Industries, Israel) at 37°C in a humidified incubator with 5% CO_2_. All cell lines were confirmed to be free of mycoplasma contamination.

#### 2.8.2 Gene knockdown experiment

To generate stable SLC43A2-knockdown cell lines, ZsGreen1-cMYC/pLVX-puromycin vectors targeting SLC43A2 were purchased from HanBio (RRID: Addgene_180278). The multiplicity of infection (MOI) was set at 30, calculated as MOI = (viral titer × viral volume)/cell number. Puromycin (Solarbio, Beijing, China) was used to screen stable cell lines for 4 weeks before subsequent experiments. Three shRNAs targeting SLC43A2 (sh#1, sh#2, sh#3) and a negative control (NC) were commercially obtained from Sangon (China). Transfection was performed using Lipofectamine 3000 (Invitrogen, USA). The shRNA sequences were as follows:

sh#1: 5′-GACCTTCGGTCCACGTTTATTCTCGAGAATAAACGTGGACCGAAGGTC -3′
sh#2: 5′-TTGCGTACGGAGCAAGTAAACTCGAGTTTACTTGCTCCGTACGCAA-3′ sh#3: 5′-CGGTGCTAATTCCTTTGTAATCTCGAGATTACAAAGGAATTAGCACCG-3′

#### 2.8.3 Experimental grouping

U937 cells were divided into knockdown and control groups, both cultured in RPMI-1640 medium (Gibco, USA) containing 10% FBS (Biological Industries, Israel).

#### 2.8.4 RNA isolation and quantitative real-time PCR

Total RNA was extracted using Trizol reagent (Invitrogen, USA) following the manufacturer’s protocol. RNA concentration was quantified using a NanoDrop™ One spectrophotometer. Subsequently, 1.5 µg of RNA was reverse-transcribed into cDNA using the Evo M-MLV RT Kit (AG, China). PCR amplification was performed using the SYBR Green PCR Mastermix Kit (AG, China) on a QuantStudio™ real-time PCR system. Quantitative analysis was conducted using the 2−ΔΔCt method. The primer sequences were:

GAPDH:
Forward: 5′-GACCTGACCTGCCGTCTAGAAA-3′
Reverse: 5′-CCTGCTTCACCACCTTCTTGA-3′.
SLC43A2:
Forward: 5′-AGTCAGAGGGCTTTTACTCCTAC-3′
Reverse: 5′-GTCCATGACGATACCCAGGG-3′

The thermal cycling conditions were as follows: Stage 1 (1 cycle): 95°C for 30 sec (polymerase activation); Stage 2 (40 cycles): 95°C for 10 sec (denaturation), 60°C for 30 sec (annealing); Stage 3 (1 cycle): Melt curve analysis (instrument default program).

#### 2.8.5 Flow cytometry analysis of tumor cell viability and apoptosis

The viability and apoptosis of U937 cells in knockdown and control groups were assessed by flow cytometry. An Annexin V-FITC Apoptosis Detection Kit (Beyotime, China) was used. Cells from both groups were collected, resuspended in 195 µL Annexin V-FITC binding buffer, and stained with 5 µL Annexin V-FITC and 10 µL propidium iodide solution. After gentle mixing, cells were incubated at room temperature in the dark for 15 min. Apoptotic cell ratios were determined using a BD FACSCanto™ II flow cytometer (USA).

#### 2.8.6 Cell co-culture model

A 1:1 effector-to-target ratio was used to co-culture the two cell groups with T cells in 6-well plates. The cytotoxic effects of T cells on both groups were evaluated at 24 h, 48 h, and 72 h. Antibody-based assays were employed to compare T-cell infiltration capacity, exhaustion marker expression, and subset distribution between the two groups.

#### 2.8.7 Statistical analysis

Intergroup differences were analyzed using t-test. Survival analysis was performed using Kaplan-Meier curves, log-rank tests, and Cox proportional hazards regression models. Correlations were assessed using Spearman’s rank correlation. A P-value < 0.05 was considered statistically significant in all analyses.

## 3 Results

### 3.1 Expression of SLC43A2 genes in AML

**Figure 1:**
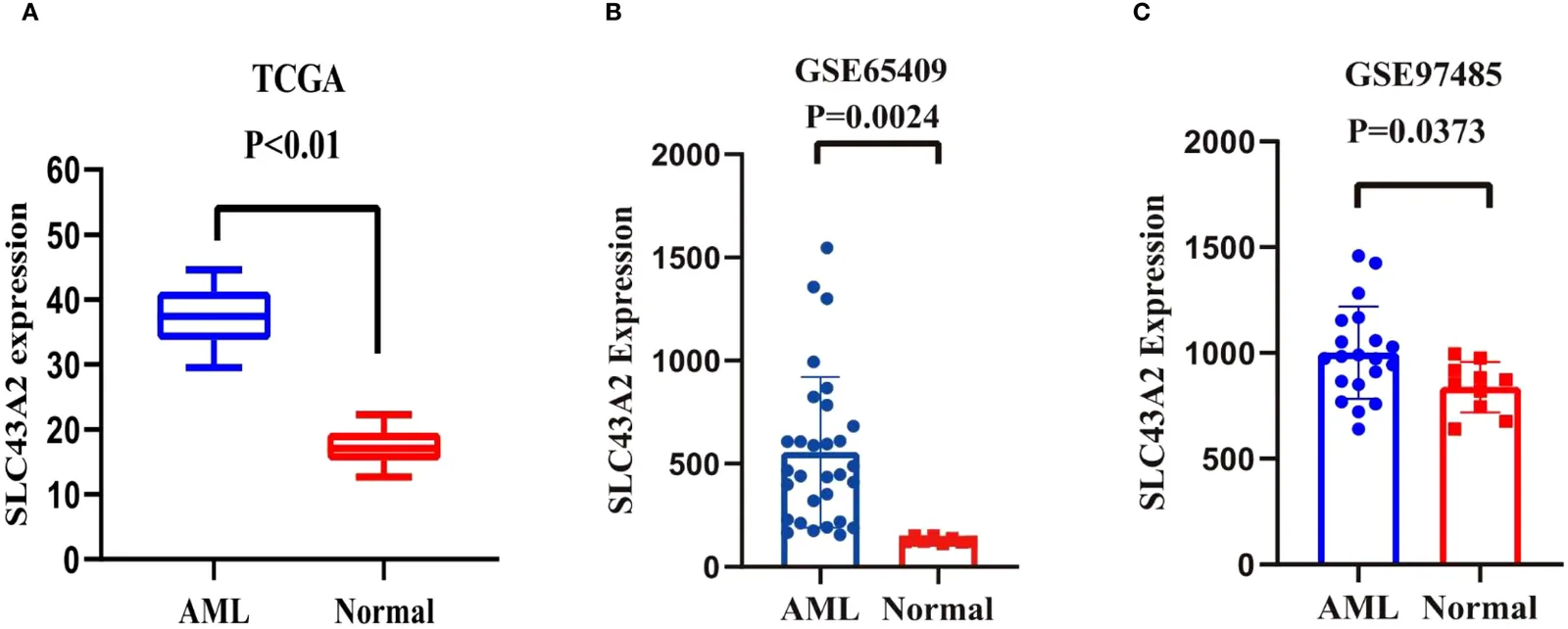
Differential expression of SLC43A2. **(A-C)** Data from TCGA datasets and two different GEO datasets showed that the expression level of SLC43A2 is significantly higher in AML compared to normal tissues (P < 0.05).

To explore the role of SLC43A2 in the pathogenesis of AML, **Figure 1A** illustrates the differential expression of SLC43A2 in the TCGA database, and the results show that the expression level of SLC43A2 is significantly higher in AML compared to normal tissues. We also evaluated the expression levels of SLC43A2 in AML patients and normal controls based on the GSE65409 and GSE97485 cohorts from the GEO database. The results showed that SLC43A2 expression was significantly higher in AML patients compared to normal controls (P < 0.01) (**Figures 1B, C**).

### 3.2 Survival analysis of SLC43A2 genes in AML

**Figure 2:**
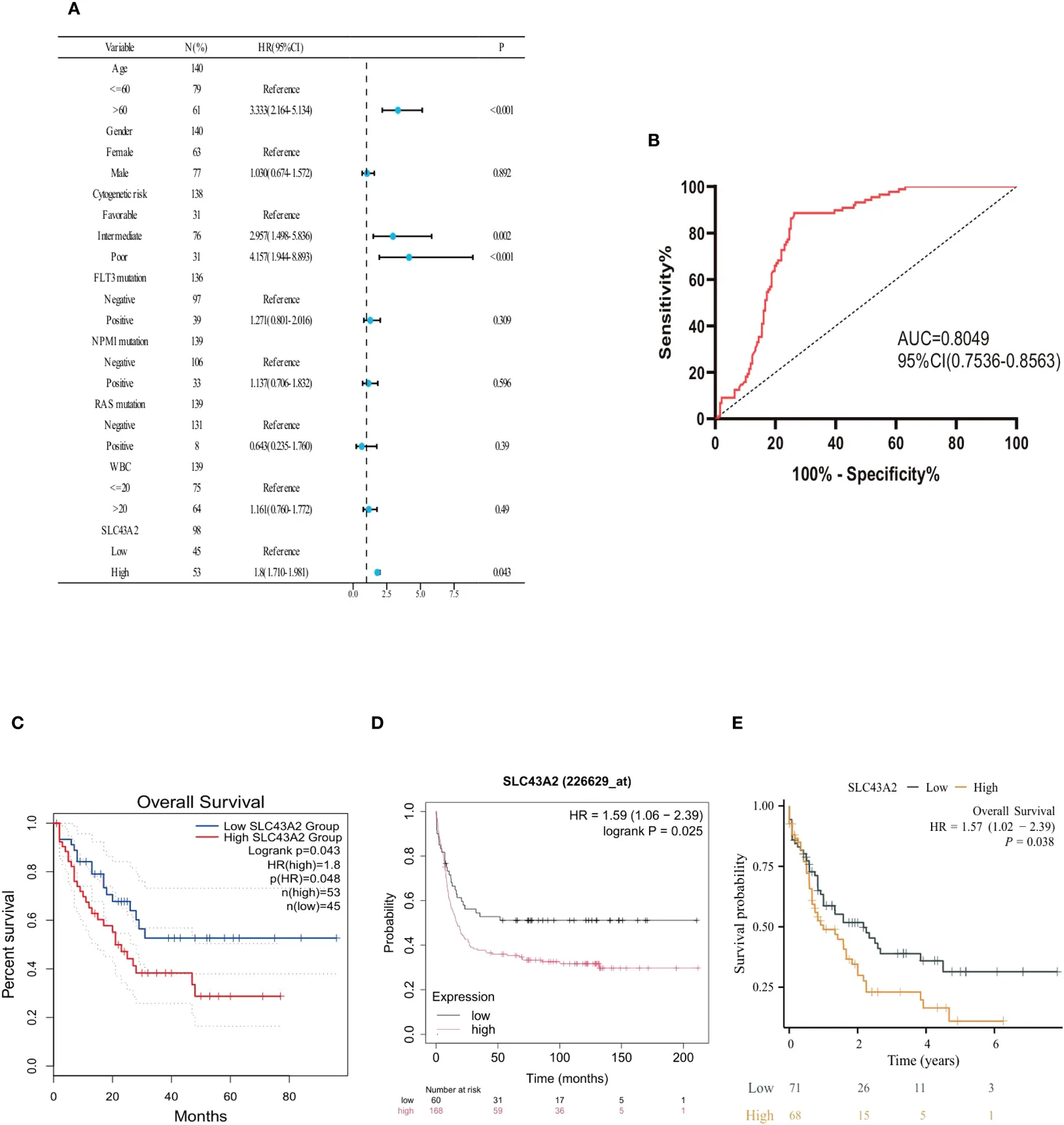
Prognostic value of SLC43A2 in AML. **(A)** Forest plot shows the results of univariate Cox regression analysis of SLC43A2 expression on overall survival (OS) in AML patients with different clinicopathological features. **(B)** ROC curve and AUC of SLC43A2 in the TCGA dataset. **(C-E)** Kaplan-Meier curves of OS in AML patients with high/low SLC43A2 expression.

To evaluate the prognostic significance of SLC43A2 in AML, we performed univariate survival analysis incorporating clinical variables such as patient age, sex, white blood cell count, cytogenetic risk, FLT3 mutation, NPM1 mutation, RAS mutation, and SLC43A2 mRNA expression level. The results indicated that age, cytogenetic risk, and SLC43A2 expression level were associated with clinical outcomes in AML patients (**Figure 2A**). The receiver operating characteristic (ROC) curve and the area under the ROC curve (AUC) were used to assess the predictive ability of SLC43A2. The ROC curve illustrates the relationship between the true positive rate (TPR) and false positive rate (FPR) for a classification model, with the AUC serving as a comprehensive measure of the model’s overall performance across different thresholds. The AUC of SLC43A2 was 0.8049 (95%CI 0.7536-0.8563) showing excellent predictive value (**Figure 2B**).

Additionally, Kaplan-Meier curves confirmed that elevated SLC43A2 expression negatively impacted overall survival (OS) in AML patients (GEPIA database analysis, HR = 1.8, p = 0.048) (**Figure 2C**). The Kaplan-Meier (K-M) Plotter database also demonstrated that high SLC43A2 expression was associated with inferior OS (HR = 1.59, P = 0.025) (**Figure 2D**). In the TCGA-LAML cohort, patients with high SLC43A2 expression had significantly lower 10-year OS (HR = 1.57, p = 0.038) (**Figure 2E**).

### 3.3 Function enrichment analysis of SLC43A2 genes in AML

**Figure 3:**
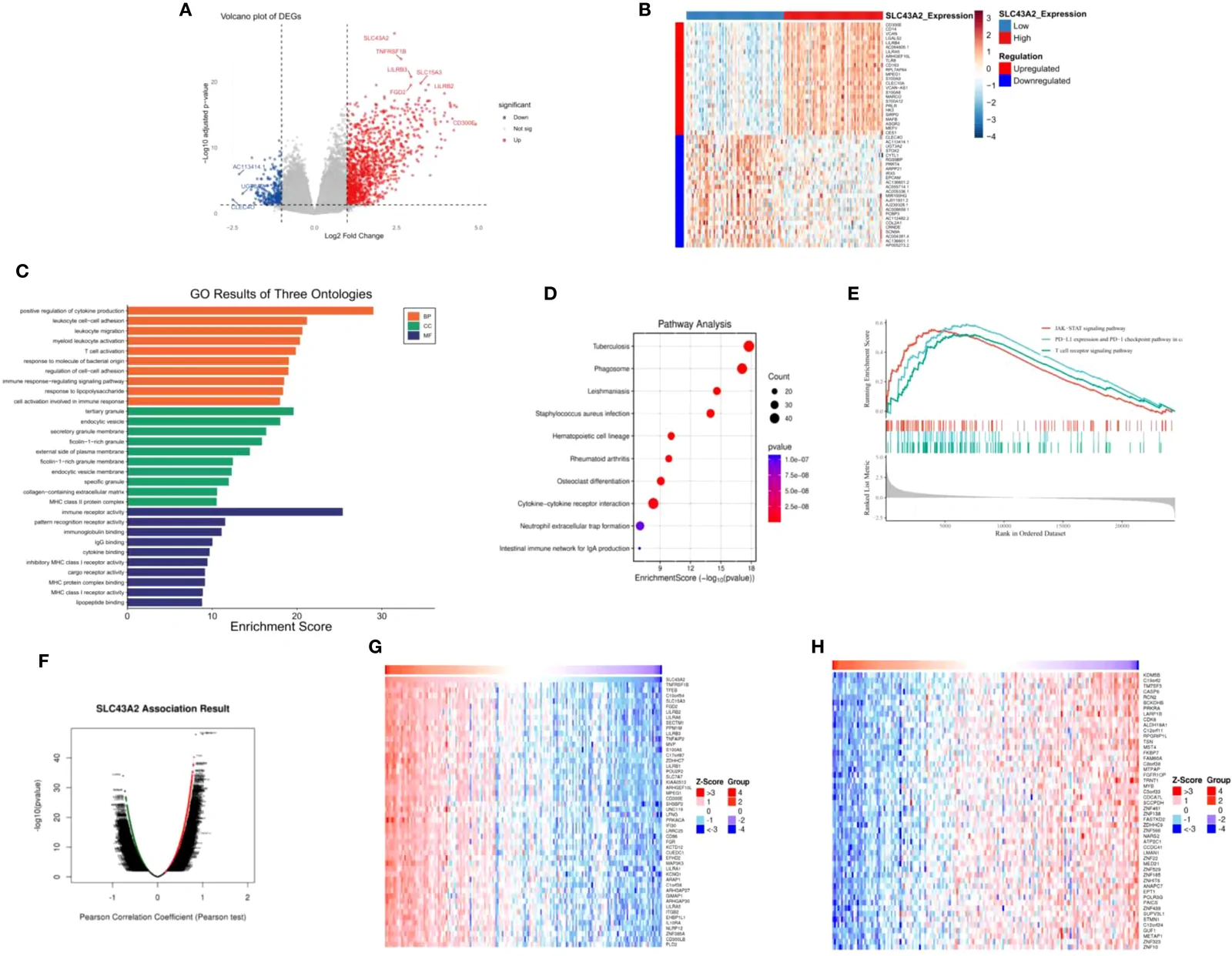
Potential mechanism of action of SLC43A2 in AML. **(A)** Volcano plot showing differential genes in AML patients with high or low SLC43A2 expression. **(B)** Heat map showing the top 20 differentially expressed genes. **(C, D)** GO and KEGG analysis of differentially expressed genes. **(E)** GSEA showed pathway enrichment in the SLC43A2 high and low expression groups. **(F)** Volcano plot showing all the related genes of SLC43A2. **(G, H)** Top 50 genes with positive/negative correlations with SLC43A2.

Differential gene expression analysis was performed between AML patients with high and low SLC43A2 expression. A total of 1124 upregulated genes and 233 downregulated genes were identified based on the criteria of p.adjust < 0.05 and |log2 FC| > 1 (**Figure 3A**). This heat map shows the top 25 genes that were significantly downregulated and significantly upregulated in the SLC43A2 low-expression group compared to the high-expression group (**Figure 3B**). GO term annotation analysis (**Figure 3C**) revealed that in the biological process (BP) category, SLC43A2 is associated with cell activation involved in immune response, immune response-regulating signaling pathway, and T cell activation. In the cellular component (CC) category, SLC43A2 is associated with collagen-containing extracellular matrix, specific granule, and endocytic vesicle membrane. In the molecular function (MF) category, SLC43A2 is associated with lipopeptide binding and MHC class I receptor activity. KEGG analysis (**Figure 3D**) indicated that SLC43A2 is involved in cytokine-cytokine receptor interaction, hematopoietic cell lineage, and neutrophil extracellular trap formation. Additionally, GSEA compared the high SLC43A2 expression group with the low SLC43A2 expression group to reveal potential mechanisms (**Figure 3E**). The high SLC43A2 expression group was enriched in the JAK-STAT signaling pathway, PD-L1 expression and PD-1 checkpoint pathway, and T cell receptor signaling pathway. Furthermore, we constructed the LinkFinder module of SLC43A2 using the LinkedOmics web portal to better understand the co-expressed genes of SLC43A2 in AML (**Figure 3F**). Heatmaps were used to display the top 50 positively correlated genes (**Figure 3G**) and negatively correlated genes (**Figure 3H**).

### 3.4 Immune infiltration analysis of SLC43A2 genes in AML

**Figure 4:**
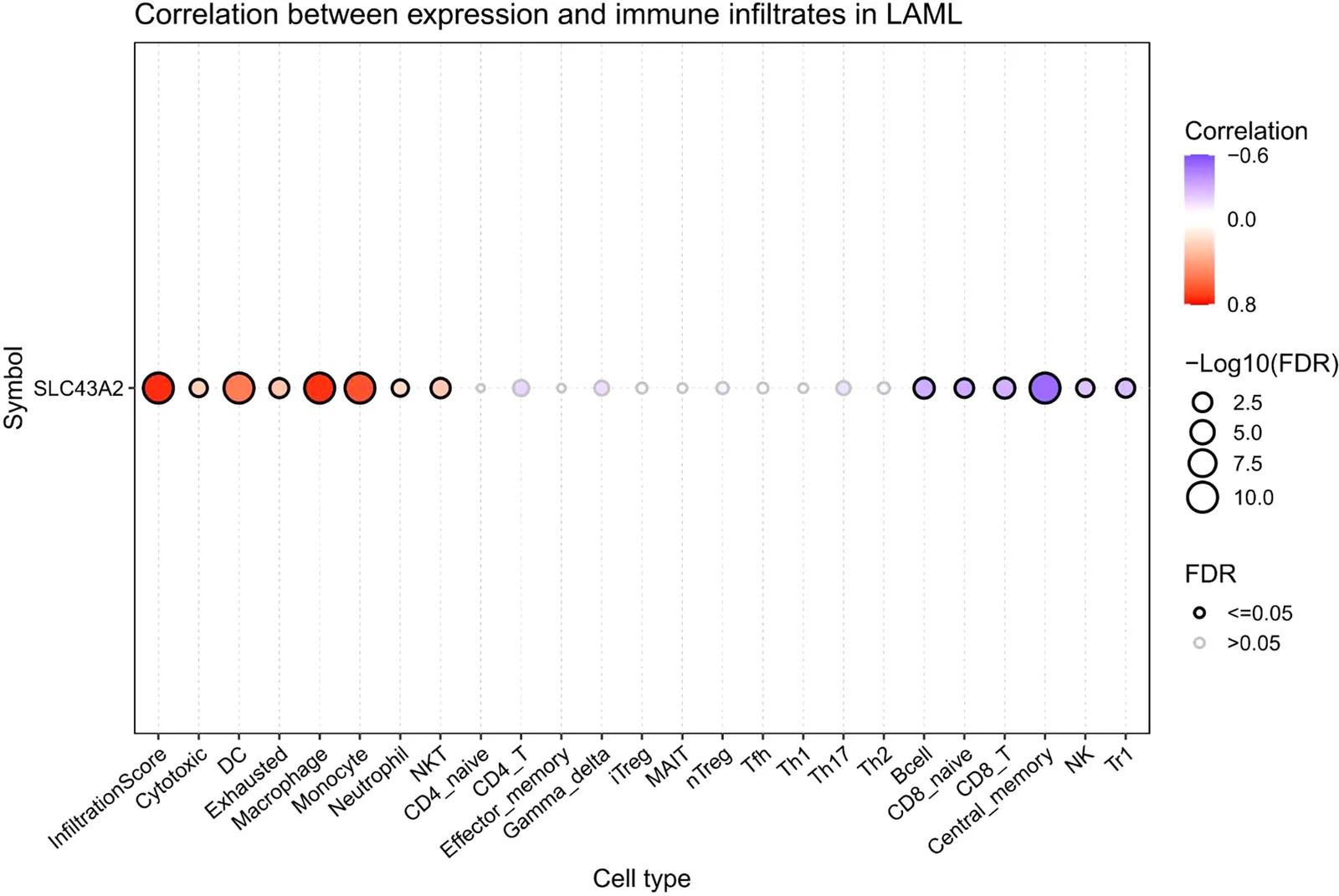
The correlation between the expression of SLC43A2 mRNA and the infiltration degree of 24 types of immune cells. SLC43A2 was positively correlated with infiltration score, cytotoxic cells, dendritic cells (DC), exhausted cells, and macrophage cells, and negatively correlated with Tr1 cells, natural killer (NK) cells, central memory cells, CD8+ T cells, and CD8+ naive cells.

Subsequently, we evaluated the correlation between SLC43A2 mRNA expression and the infiltration levels of 24 immune cell types. As shown in **Figure 4**, the SLC43A2 gene was significantly associated with multiple immune cell types. Specifically, SLC43A2 was positively correlated with infiltration score, cytotoxic cells, dendritic cells (DC), exhausted cells, and macrophage cells, and negatively correlated with Tr1 cells, natural killer (NK) cells, central memory cells, CD8+ T cells, and CD8+ naive cells (**Supplementary Figures S1**, **S2**). These data suggest that SLC43A2 may exert an important immunomodulatory role in AML by influencing the infiltration of immune cells.

### 3.5 Correlation analysis between SLC43A2 genes and immune checkpoints in AML

**Figure 5:**
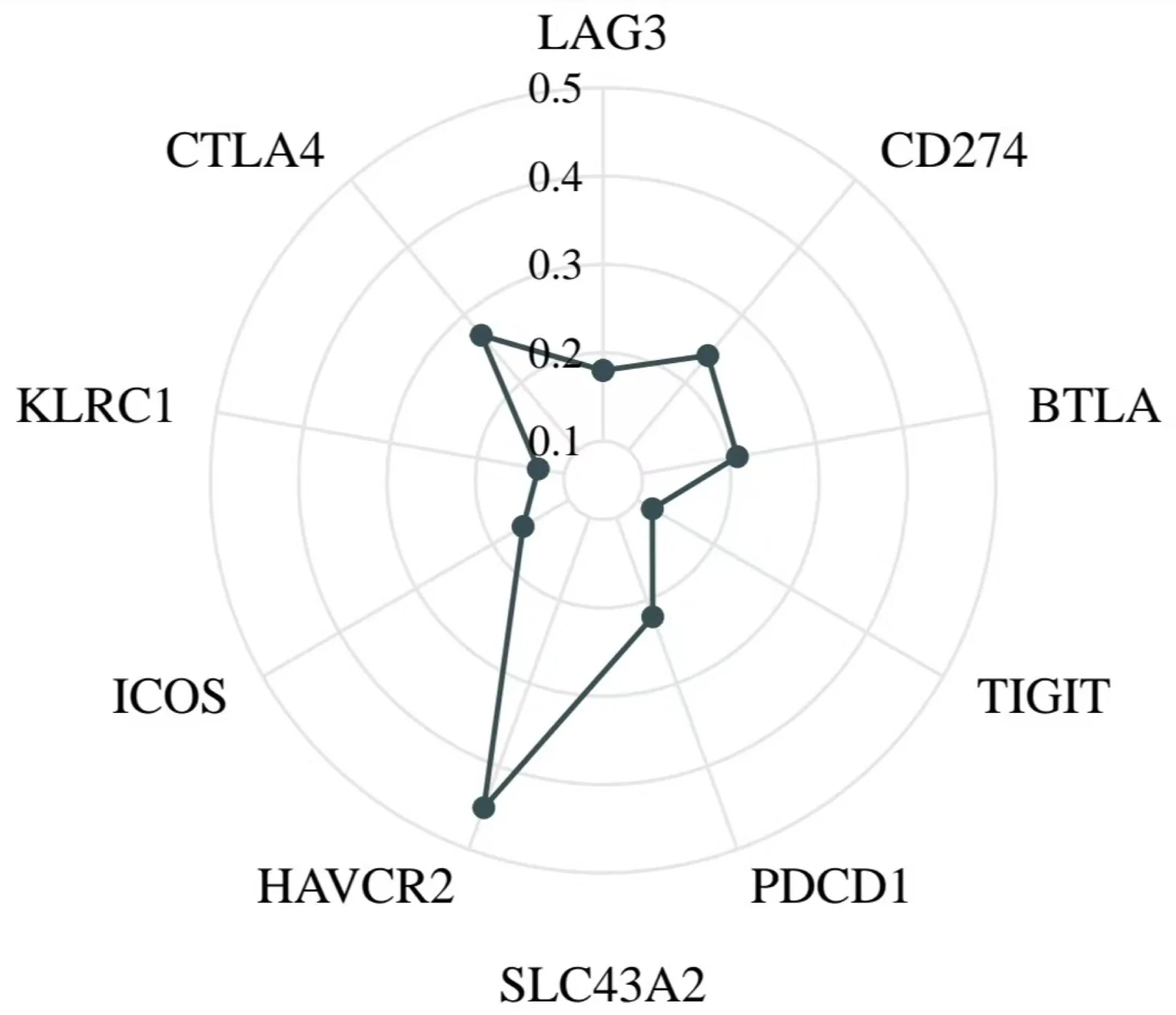
Relationship between SLC43A2 expression and immune checkpoints. SLC43A2 expression was significantly positively correlated with BTLA (r = 0.21), CTLA4 (r = 0.27), CD274 (r = 0.24), LAG3 (r = 0.18), TIGIT (r = 0.12), KLRC1 (r = 0.13), ICOS (r = 0.16), and HAVCR2 (r = 0.45).

Given the crucial role of immune checkpoint molecules in tumor immune responses, we investigated the relationship between SLC43A2 expression levels and immune checkpoint expression using data from TCGA database. We analyzed several well-known immune checkpoints, including PDCD1 (PD-1), BTLA, CTLA4, CD274 (PD-L1), LAG3, TIGIT, KLRC1, ICOS, and HAVCR2 (TIM-3) (**Supplementary Figure S3**). The results showed that SLC43A2 expression was significantly positively correlated with BTLA (r = 0.21), CTLA4 (r = 0.27), CD274 (r = 0.24), LAG3 (r = 0.18), TIGIT (r = 0.12), KLRC1 (r = 0.13), ICOS (r = 0.16), and HAVCR2 (r = 0.45) (all P values < 0.001) (**Figure 5**).

### 3.6 Construction of the upstream regulatory network of SLC43A2

**Figure 6:**
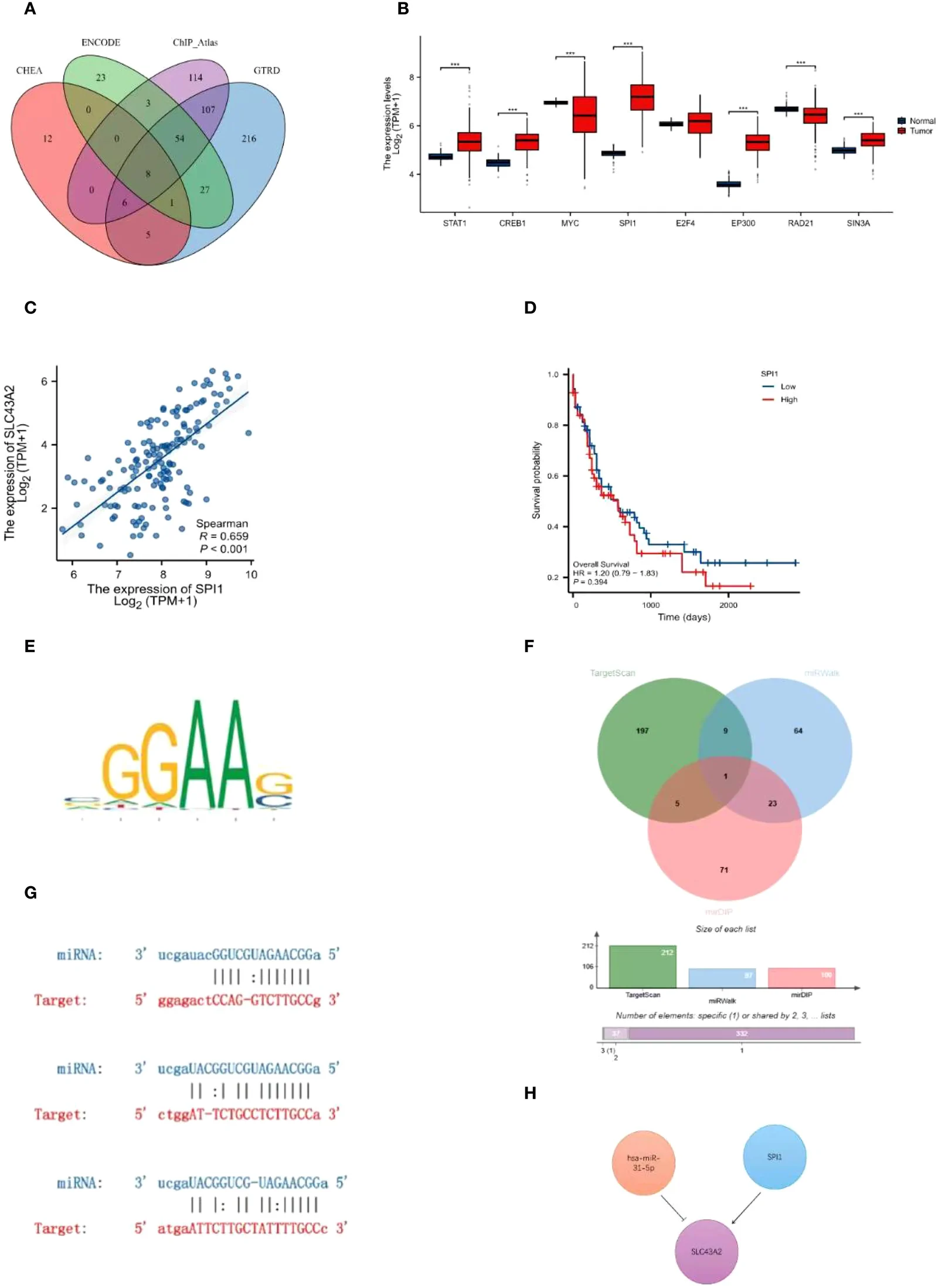
Construction of the upstream regulatory network of SLC43A2. **(A)** Venn diagram analysis identified STAT1, CREB1, MYC, SPI1, E2F4, EP300, RAD21 and SIN3A as potential TFS of SLC43A2. **(B)** Expression levels of STAT1, CREB1, MYC, SPI1, E2F4, EP300, RAD21 and SIN3A in TCGA-LAML dataset. **(C)** Scatter plot of the association between SPI1 and SLC43A2. **(D)** Comparison of OS between SPI1 high and low expression groups. **(E)** Predicted transcription factor binding motifs. **(F)** Venn diagram analysis identified hsa-miR-31-5p as a potential miRNA of SLC43A2. **(G)** Predicted interaction between SLC43A2 and hsa-miR-31-5p. **(H)** Potential upstream TF-miRNA-mRNA regulatory network of SLC43A2. ***P<0.001.

We investigated the upstream regulatory mechanisms of SLC43A2 by identifying potential TFs and miRNAs that may target SLC43A2. Candidate TFs were screened from the CHEA, GTRD, ENCODE, and ChIP_Atlas databases. By intersecting these databases, we identified eight key TFs that are most likely to target SLC43A2 (**Figure 6A**). We then analyzed the expression levels of these eight potential TFs in AML (**Figure 6B**) and their correlation with SLC43A2 (**Supplementary Figure S4**). The results showed that SPI1 was upregulated in AML and exhibited the highest correlation with SLC43A2 (r = 0.659, P < 0.001) (**Figure 6C**). Survival analysis showed that there was no significant difference in OS between SPI1 high and low expression groups (**Figure 6D**). The predicted TF binding sites are illustrated in **Figure 6E**. Subsequently, we explored the upstream miRNA regulatory factors of SLC43A2 using miRWalk, mirDIP, and TargetScan. First, we obtained the predicted miRNAs by intersecting these three datasets (**Figure 6F**). The results indicated that hsa-miR-31-5p is the most likely miRNA involved in the post-transcriptional regulation of SLC43A2. We also demonstrated the complementary sequence between SLC43A2 and hsa-miR-31-5p (**Figure 6G**). Based on these findings, we constructed a transcriptional network involving SPI1, hsa-miR-31-5p, and SLC43A2 (**Figure 6H**).

### 3.7 Mutational analysis of SLC43A2 related genes

**Figure 7:**
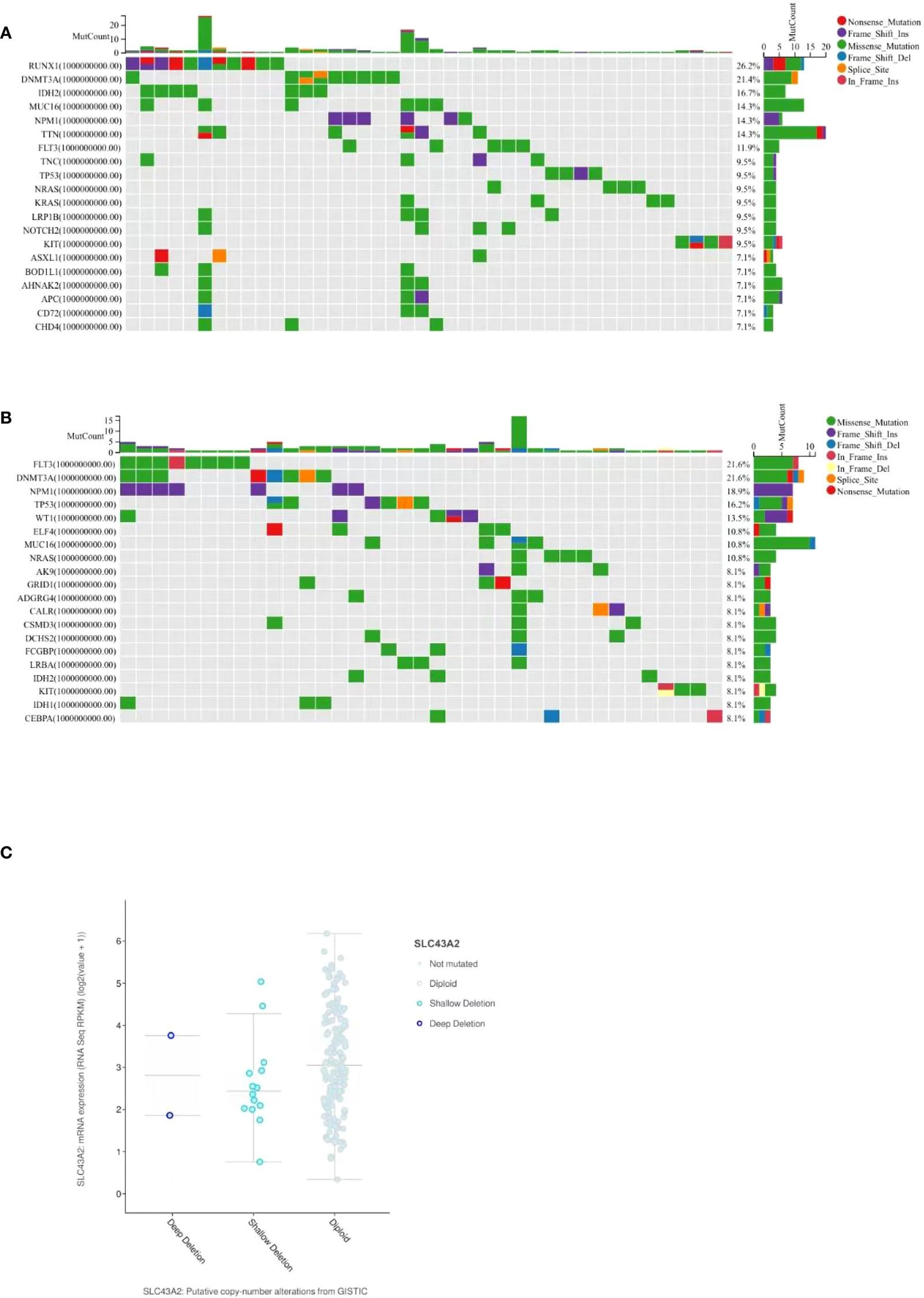
Mutational analysis of SLC43A2 related genes. **(A)** Top 20 significantly different somatic mutations in the SLC43A2 high expression group. **(B)** Top 20 significantly different somatic mutations in the SLC43A2 low expression group. **(C)** cBioPortal analysis based on TCGA-LAML data showed that deletion, diploidy, copy number gain and amplification were involved in the dysregulation of SLC43A2 expression.

AML is a severe cancer characterized by high heterogeneity and genetic factors. We analyzed the association between SLC43A2 expression and somatic mutations using data from the TCGA-LAML cohort. **Figures 7A, B** show the top 20 significantly different somatic mutations in the SLC43A2 high and low expression groups, respectively. The results indicated that mutations in RUNX1, DNMT3A, IDH2, MUC16, and NPM1 were more frequent in the high SLC43A2 expression group. These mutated genes are known biomarkers of AML and hold significant value for assessing tumor malignancy progression or treatment response. Notably, deep deletions and diploid mutations of SLC43A2 were relatively common in TCGA-LAML tissues (**Figure 7C**).

### 3.8 Gene and protein network of SLC43A2

**Figure 8:**
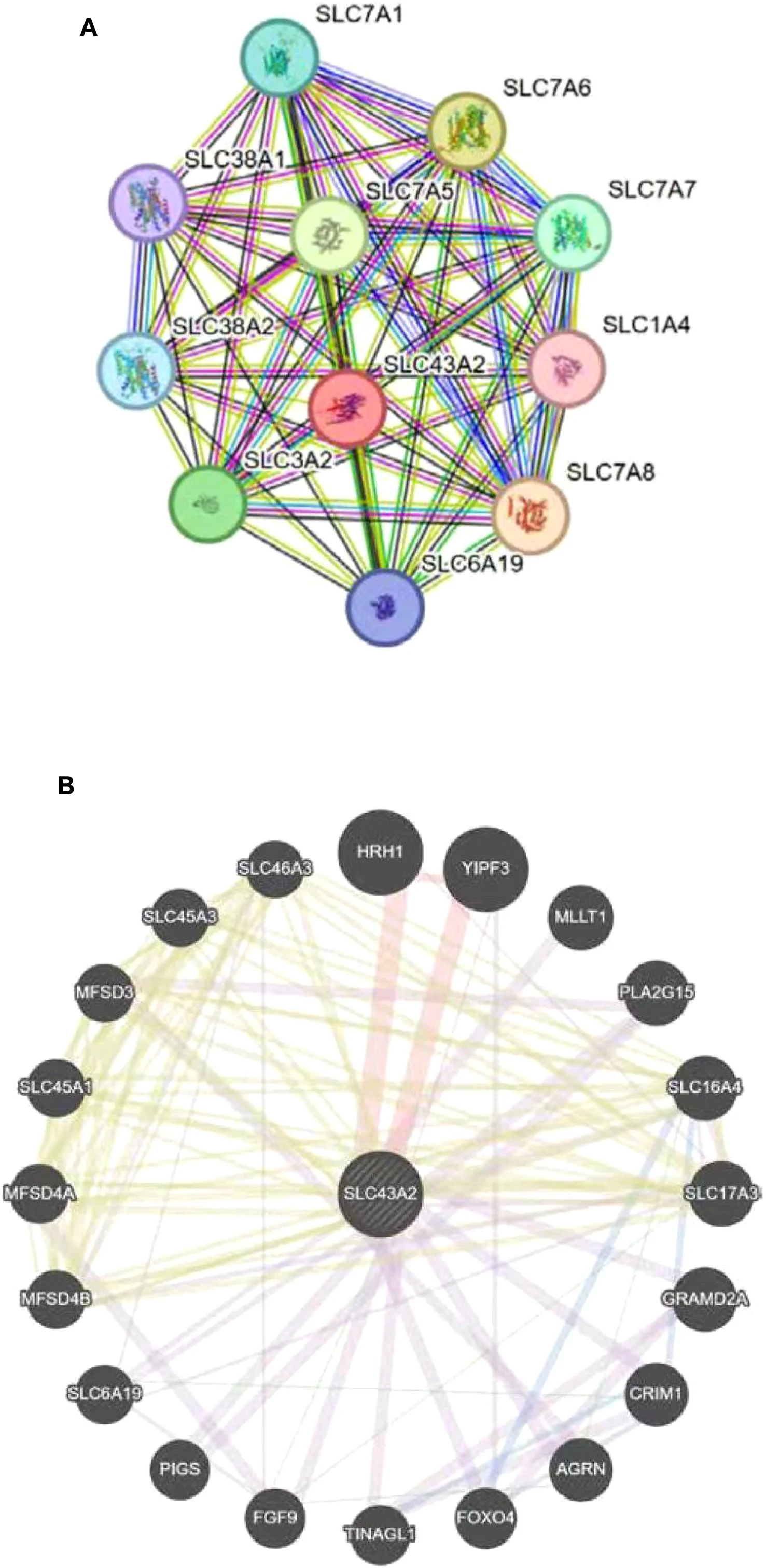
Gene and protein network of SLC43A2. **(A)** Network diagram of interactions between proteins encoded by the SLC43A2 gene. **(B)** Analysis of SLC43A2-related gene networks by GeneMANIA database.

PPI network of SLC43A2 was constructed using the STRING database. The results showed that SLC43A2 is closely related to the SLC family, such as having strong associations with SLC7A5, SLC6A19, and SLC7A1 (**Figure 8A**). Additionally, the gene–gene network of SLC43A2 was constructed using GeneMANIA. **Figure 8B** illustrates the interactions between SLC43A2 and 20 potential target genes.

### 3.9 Validation of the expression level of SLC43A2 and the correlation with immune-related markers based on vitro experiment

**Figure 9:**
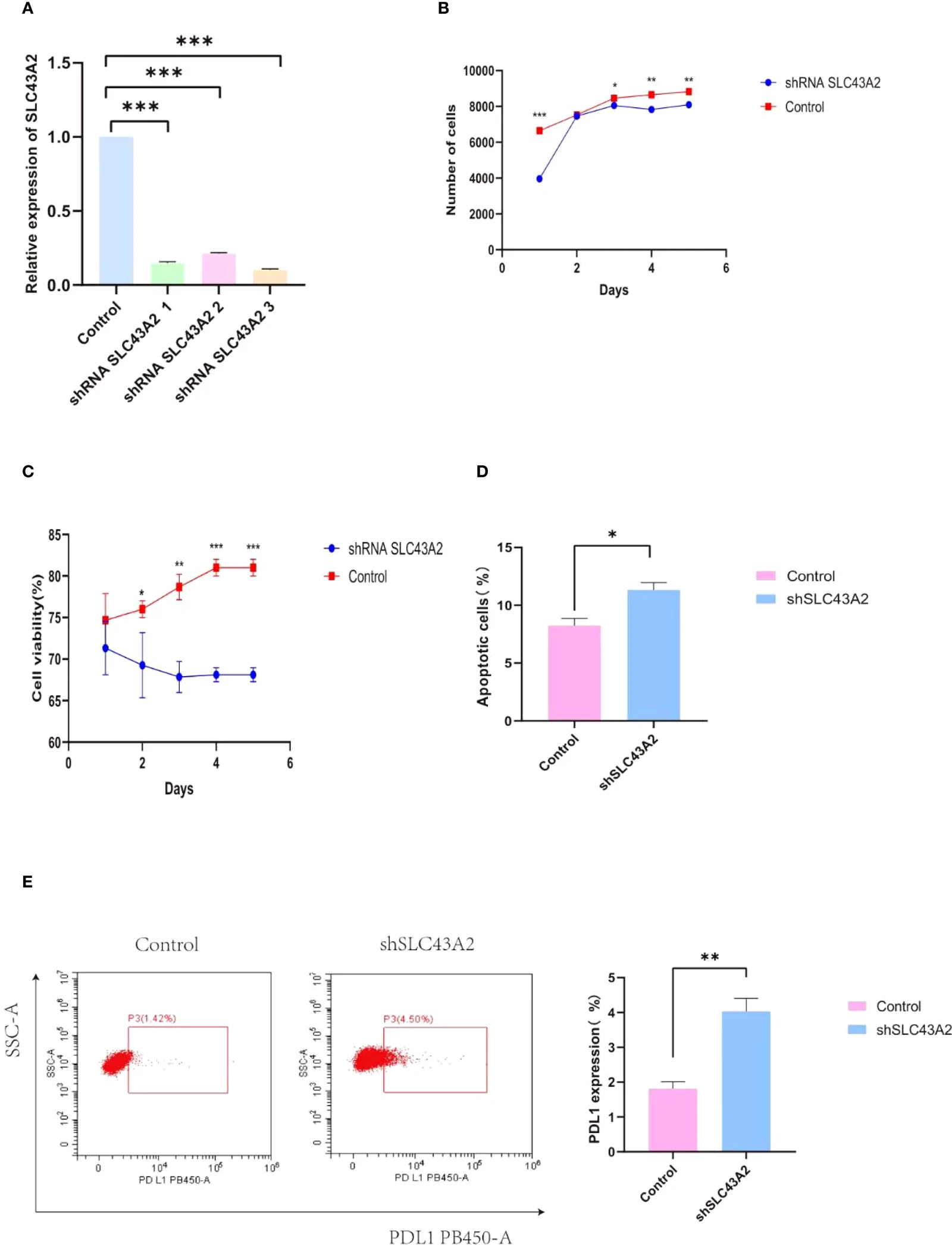
Verify the effect of SLC43A2 on tumor cells through *in vitro* experiments. **(A)** The knockdown efficiency of shRNA was verified by PCR. **(B, C)** The number and activity of U937 between the two groups were measured by flow cytometry. **(D, E)** Flow cytometry analysis showed that relatively high SLC43A2 expression promoted U937 apoptosis as well as PD-L1 expression. *P<0.05;**P<0.01;***P<0.001.

To verify the effect of SLC43A2 on tumor cells, we significantly reduced the mRNA expression level of SLC43A2 in the U937 cell line through shRNA-mediated knockdown (**Figure 9A**). Among the three shRNAs tested, shRNA3 exhibited the highest knockdown efficiency and was therefore selected for subsequent experiments. Cell number and viability were monitored in both groups using flow cytometry, and the results showed that cell number and viability was significantly inhibited over time by the knockdown of SLC43A2 (**Figures 9B, C**). Flow cytometric analysis revealed that SLC43A2 knockdown significantly increased the proportion of tumor cell apoptosis (**Figure 9D**) and the expression of PD-L1 (**Figure 9E**).

**Figure 10:**
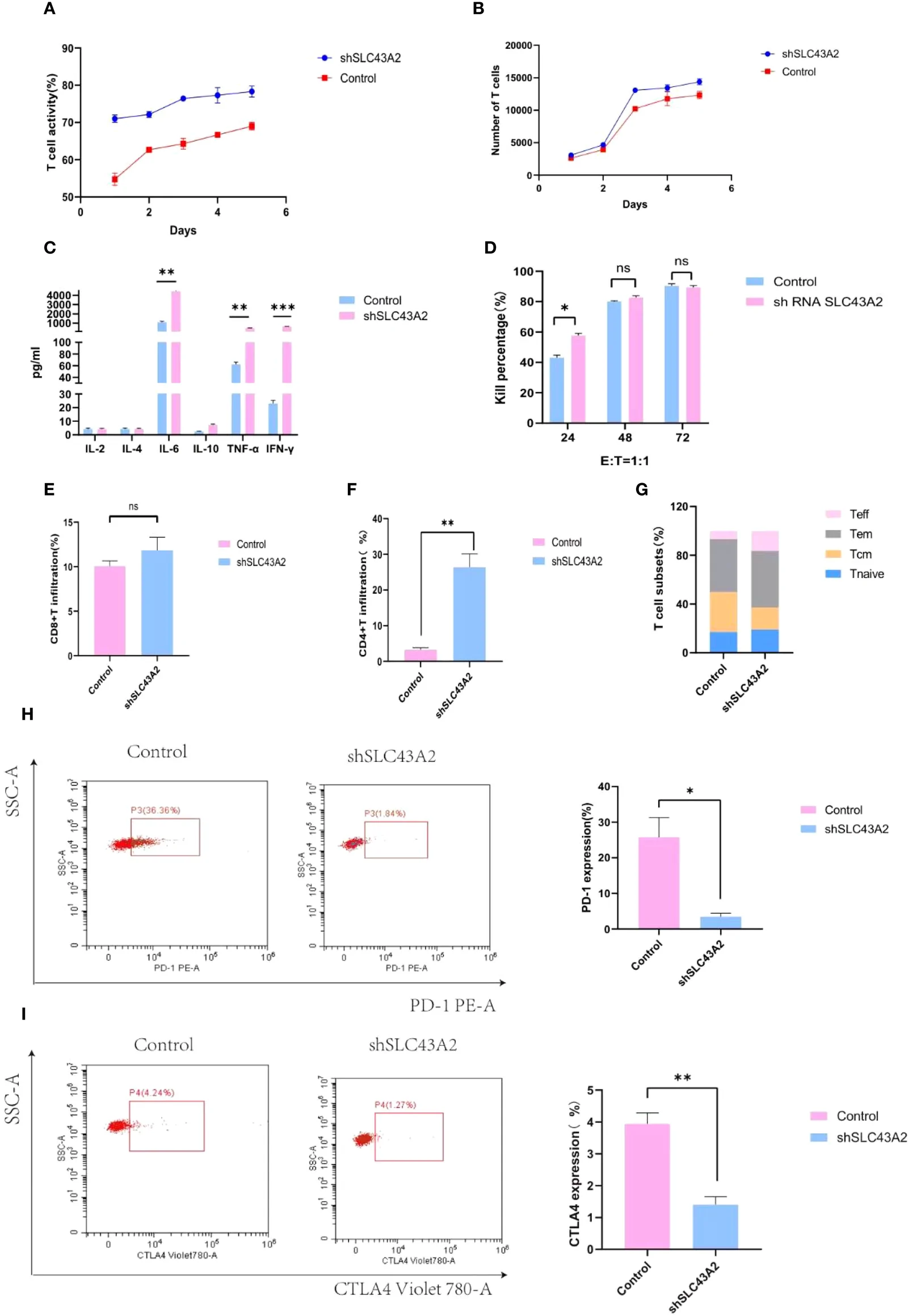
Verify the effect of SLC43A2 on T cells through *in vitro* experiments. **(A, B)** Activity and number of T cells in knockdown and control groups. **(C)** Comparison of cytokines released by T cells between control and knockdown groups. **(D)** Comparison of killing rate between control group and knockdown group. **(E)** Proportion of CD8+T infiltration in control and knockdown groups. **(F)** Proportion of CD4+T infiltration in control and knockdown groups. **(G)** Comparison of T cell subsets between control group and knockdown group. **(H, I)** Comparison of PD-1 and CTLA-4 expression in T cells between control and knockdown groups. *P<0.05;**P<0.01;***P<0.001.

To verify the effect of SLC43A2 on T cells, we co-cultured the two groups of U937 cells with T cells at an effector-to-target ratio of 1:1. Compared with the control group, the T cells in the knockdown group had higher activity and quantity (**Figures 10A, B**) and released more IL-6, TNF-α and IFN-γ (**Figure 10C**). The cytotoxicity assay results indicated that the cytotoxicity of T cells in the knockdown group was significantly enhanced at 24 hours, while no significant differences were observed at 48 and 72 hours (**Figure 10D**). Additionally, we found that there was no significant change in the infiltration of CD8+T cells (**Figure 10E**), while SLC43A2 knockdown significantly enhanced the infiltration of CD4+T cells (**Figure 10F**). T cell subset analysis showed that SLC43A2 knockdown in tumor cells significantly promoted the differentiation of effector T cells (**Figure 10G**). Compared with the control group, SLC43A2 knockdown in tumor cells significantly decreased the expression of T cell exhaustion markers, such as PD-1 (**Figure 10H**) and CTLA-4 (**Figure 10I**).

## 4 Discussion

AML is a hematologic malignancy characterized by the rapid proliferation of abnormal myeloid cells in the bone marrow, thereby impairing the production of healthy blood cells (19–21). In recent years, immunotherapy has emerged as a novel therapeutic approach for hematologic malignancies and has achieved significant progress (22–24). Tumor cells outcompete T cells for methionine by highly expressing the methionine transporter SLC43A2 (15). Given that SLC43A2 is highly expressed in various human and mouse tumor cells with different genetic backgrounds, inhibiting tumor SLC43A2 can restore methionine metabolism in effector T cells and rescue their function. However, the impact of SLC43A2 on the tumor immune microenvironment, immune-related genes, and prognosis in AML has not been reported.

We analyzed the potential associations between tumor SLC43A2, T cell characteristics, and clinical outcomes in cancer patients. Based on the TCGA database, we found that the transcriptional level of SLC43A2 was significantly higher in tumor tissues than in adjacent normal tissues. Moreover, patients with high SLC43A2 expression had poorer survival rates. Several studies have demonstrated that SLC43A2 is associated with the occurrence and development of various tumors. SLC43A2 regulates esophageal squamous cell carcinoma proliferation and apoptosis by modulating methionine metabolic activity (14). Studies have also shown that cancerous SLC43A2 alters T cell methionine metabolism and histone methylation (15). SLC43A2 may influence the prognosis of hepatocellular carcinoma by creating an immunosuppressive tumor microenvironment and modulating related immune-related genes (13). Another study has also indicated that SLC43A2 plays a key role in maintaining the growth of hepatocellular carcinoma and supports the proliferation of hepatocellular carcinoma through the mTORC1 signaling pathway (25). Therefore, establishing the role of SLC43A2 inhibition in cancer therapy is of vital importance. Consistent with previous studies, our *in vitro* experiments showed that SLC43A2 knockdown inhibited cell proliferation and migration in AML. After stratifying patients into high and low SLC43A2 expression groups, GSEA analysis revealed that the JAK-STAT signaling pathway, PD-L1 expression and PD-1 checkpoint pathway, and T cell receptor signaling pathway were significantly enriched in AML cells with high SLC43A2 expression. Additionally, SLC43A2 knockdown reduced the expression of PD-L1, an immune checkpoint gene that promotes immune evasion by inhibiting antitumor immune responses. These findings suggest that SLC43A2 may promote the development and progression of AML by regulating the cell cycle, epithelial-mesenchymal transition, and immune responses.

Recently, the dissemination and efficacy of targeted immunotherapy have begun to alter the management of cancer (26, 27). Given the intricate interplay between the tumor immune microenvironment and host immunity, the identification of predictive biomarkers is essential for personalized treatment (28). It is well established that the imbalance of T cell subsets is a common occurrence in cancer patients (29). Our findings demonstrate that the expression level of SLC43A2 is positively correlated with the infiltration of T cells. Knockdown of SLC43A2 in tumor cells significantly enhances the differentiation of effector T cells. High expression of SLC43A2 in tumors is closely associated with attenuated T cell immune responses in cancer patients. SLC43A2 knockdown may help reverse the immunosuppressive state, thereby inhibiting tumor growth. Additionally, SLC43A2 is positively correlated with several immune molecules, such as BTLA, CTLA4, CD274, LAG3, TIGIT, KLRC1, ICOS, and HAVCR2. Our experiments also revealed that the expression level of SLC43A2 is positively correlated with PDCD1 and CTLA4 in T cells. Studies have indicated that methionine metabolism regulates the expression of PDL1 in squamous cell carcinoma cells via the SLC43A2-NFκB signaling pathway (14). Dual immune checkpoint inhibitors using anti-PD-1/PD-L1 and anti-CTLA4 monoclonal antibodies are being extensively evaluated for tumor therapy. The combination of nivolumab (anti-PD-1 IgG4) and ipilimumab (fully human anti-CTLA4 IgG1) is one of the most popular immunotherapy regimens currently in use (30, 31). The combination of immune checkpoint inhibitors may represent a novel strategy for tumor treatment (32).

Finally, this study has several major highlights. In addition to being discovered in the TCGA dataset, the expression of SLC43A2 was also validated in the GEO database and PCR analysis, which makes our results more reliable. Furthermore, we have determined the correlation between SLC43A2 and immunity from multiple perspectives. Most importantly, this is the first study to investigate the prognostic and immunological roles of SLC43A2 in the occurrence and progression of AML. There are also some limitations in this study. Firstly, there is a lack of clinical information. In addition to tumor biology, there are several other factors that can affect the prognosis of AML patients, including clinical medical data related to their treatment centers. Therefore, the role of the SLC43A2 gene has not yet been fully studied. Secondly, the mechanism of action of SLC43A2 in AML needs to be verified *in vivo*. We will address these limitations in future research. Thirdly, although our analysis revealed a significant association between SLC43A2 expression and immune cell infiltration/checkpoint markers, the tumor microenvironment is a complex ecosystem, and the observed correlations may reflect indirect relationships or a common dependence on other factors. Future studies using gene manipulation models are needed to conduct mechanism research and establish causality.

## 5 Conclusion

The SLC43A2 gene may serve as a diagnostic, prognostic, and potential immune-related biomarker for AML patients. Blocking SLC43A2-associated signaling pathways could provide novel insights into immunotherapy for AML.

## Data Availability

The datasets presented in this study can be found in online repositories. The names of the repository/repositories and accession number(s) can be found in the article/**Supplementary Material**.

## Supplementary material

Supplementary Figure 1 Immune infiltration analysis of SLC43A2 genes in AML.SLC43A2 was positively correlated with infiltration score **(A)**, cytotoxic cells **(B)**, dendritic cells **(C)**, exhausted cells **(D)**, and macrophage cells **(E)**, monocyte infiltration score **(F)** and neutrophil infiltration score **(G)**.

Supplementary Figure 2 Immune infiltration analysis of SLC43A2 genes in AML.SLC43A2 was negatively correlated with CD4+ T cells **(A)**, CD8+ T cells **(B)**, B cells **(C)** Tr1 cells **(D)**, central memory cells **(E)**, CD8+ naive cells **(F)**, and natural killer cells **(G)**.

Supplementary Figure 3 Correlation analysis between SLC43A2 genes and immune checkpoints in AML, including BTLA **(A)**, CD274 **(B)**, CTLA4 **(C)**, HAVCR2 **(D)**, ICOS **(E)**, KLRC1 **(F)**, LAG3 **(G)**, PDCD1 **(H)** and TIGIT **(I)**.

Supplementary Figure 4 TFs in AML and their correlation with SLC43A2, including STAT1 **(A),** CREB1 **(B)**, MYC **(C),** E2F4 **(D)**, EP300 **(E)**, RAD21 **(F)** and SIN3A **(G)**.
