# Supplementary figures and images for: SLC43A2-mediated immune cell infiltration: a potential therapeutic target in acute myeloid leukemia
Source: Front Immunol. 2025 Sep 25;16:1655766. doi: 10.3389/fimmu.2025.1655766 (PMC12507577; doi:10.3389/fimmu.2025.1655766)

A

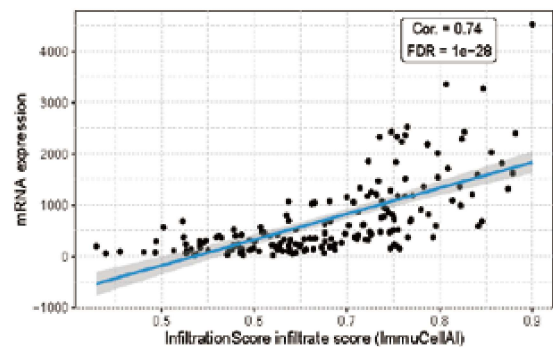

B

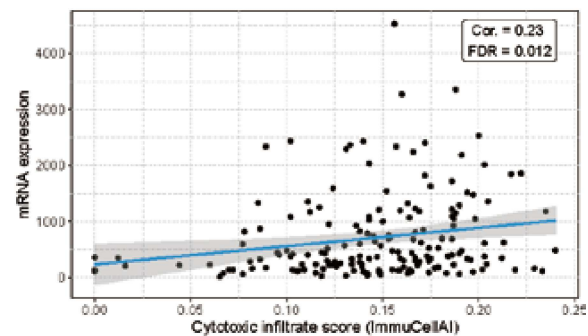

C

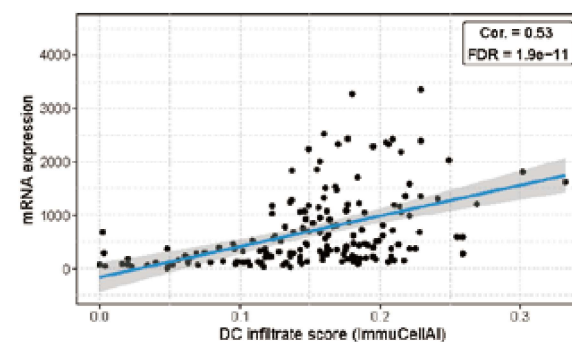

D

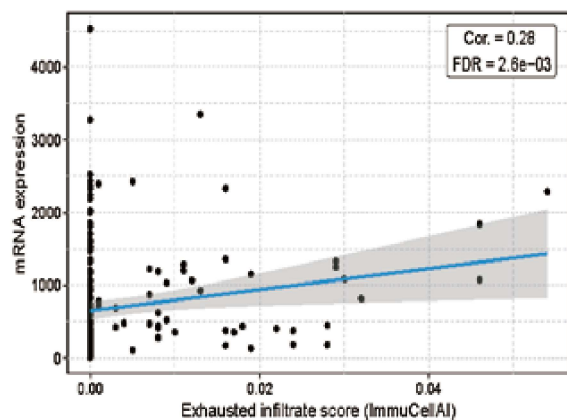

E

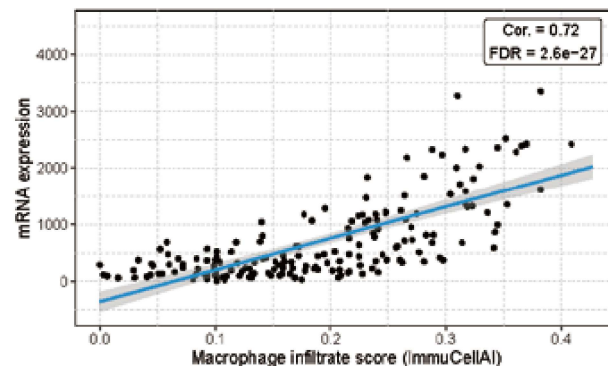

F

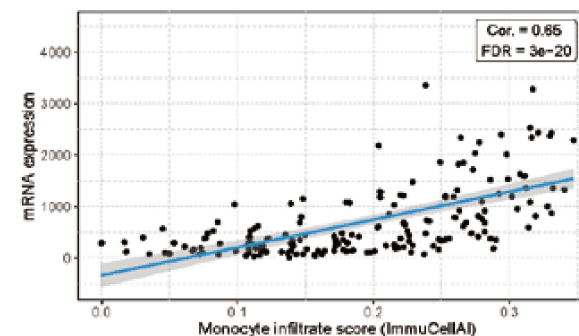

G

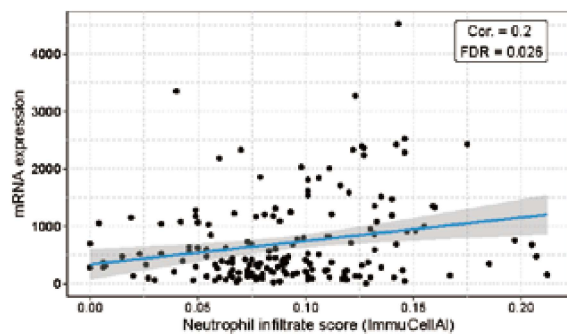

Supplement: Supplementary Figure 1 — Immune infiltration analysis of SLC43A2 genes in AML.SLC43A2 was positively correlated with infiltration score (A), cytotoxic cells (B), dendritic cells (C), exhausted cells (D), and macrophage cells (E), monocyte infiltration score (F) and neutrophil infiltration score (G). [file DataSheet1.zip › Supplementary Figure 1.pdf]

A

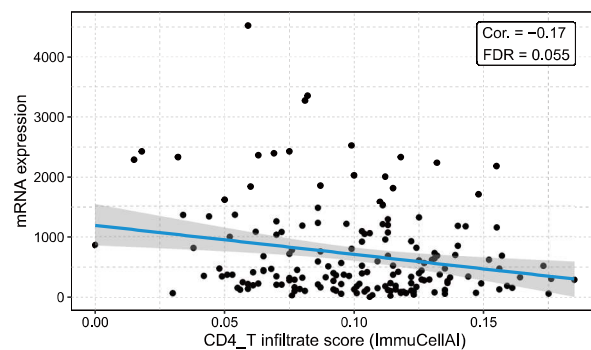

B

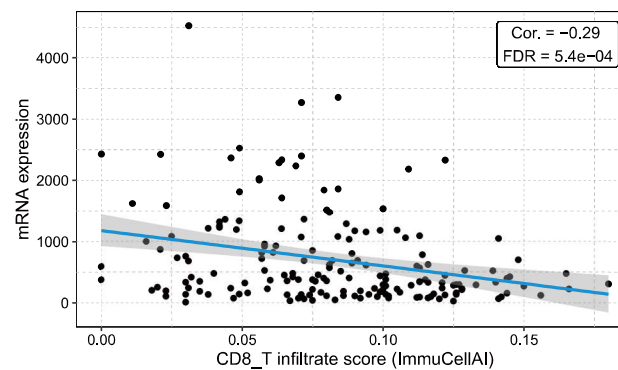

C

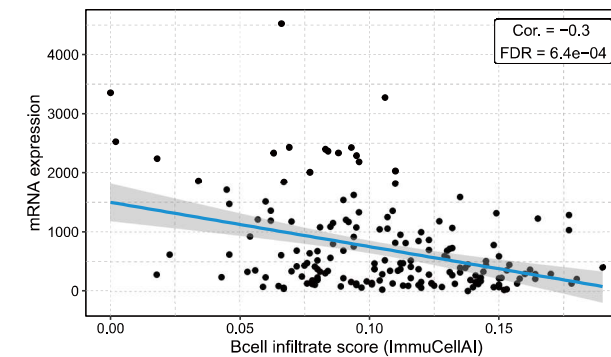

D

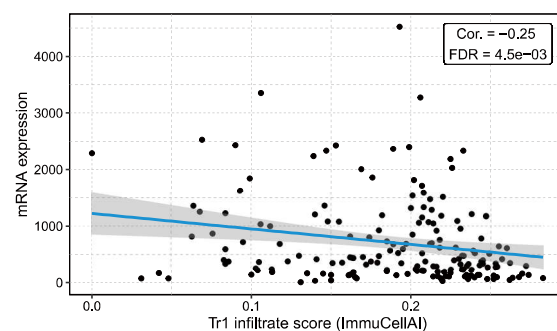

F

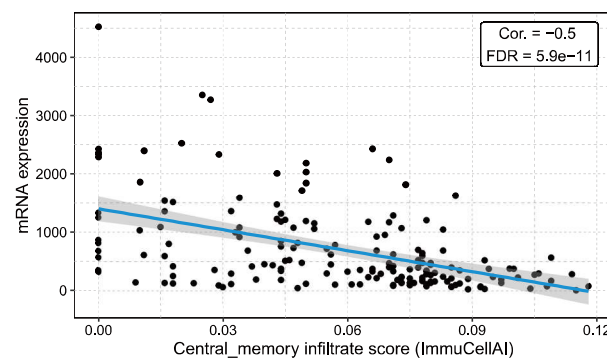

G

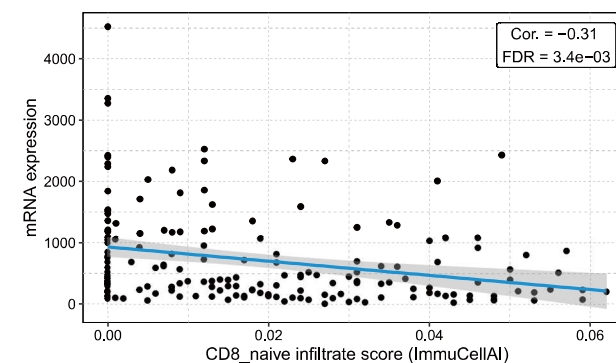

H

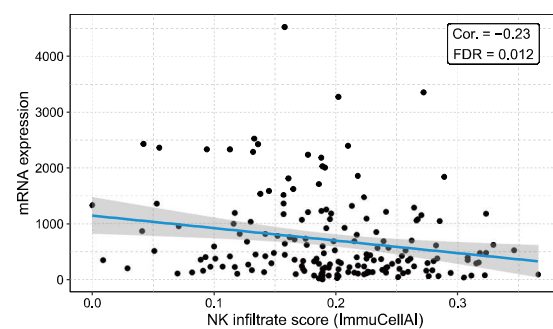

Supplement: Supplementary Figure 1 — Immune infiltration analysis of SLC43A2 genes in AML.SLC43A2 was positively correlated with infiltration score (A), cytotoxic cells (B), dendritic cells (C), exhausted cells (D), and macrophage cells (E), monocyte infiltration score (F) and neutrophil infiltration score (G). [file DataSheet1.zip › Supplementary Figure 2.pdf]

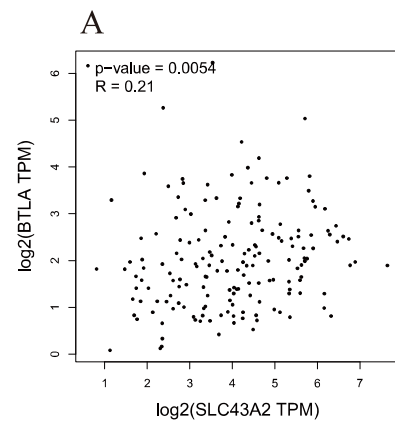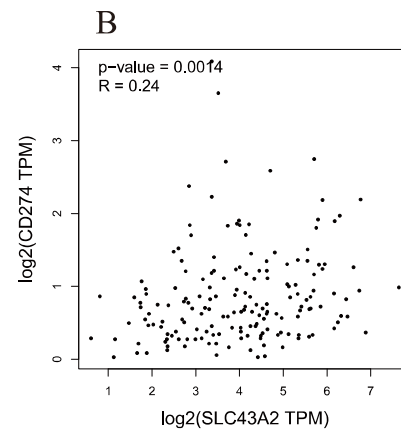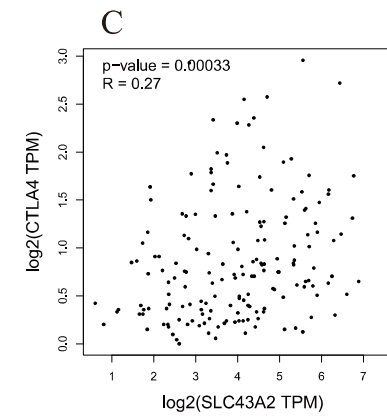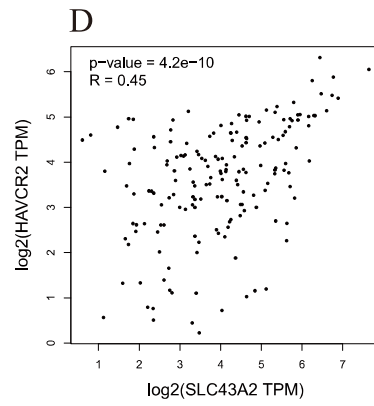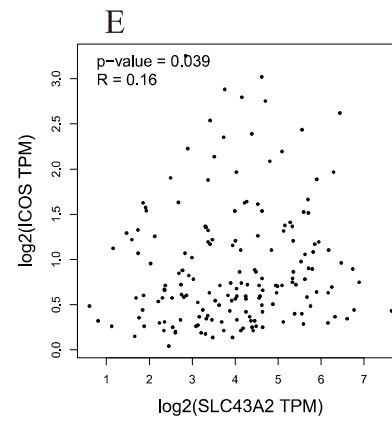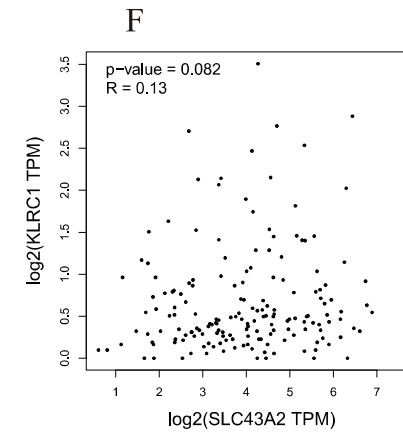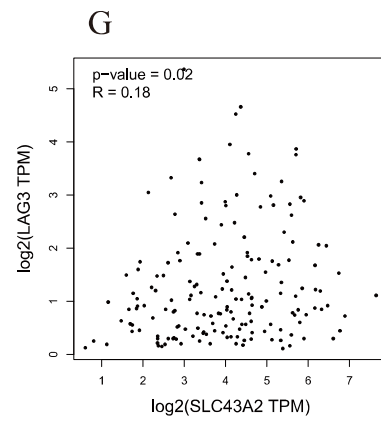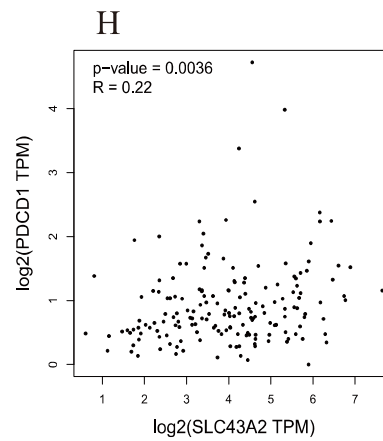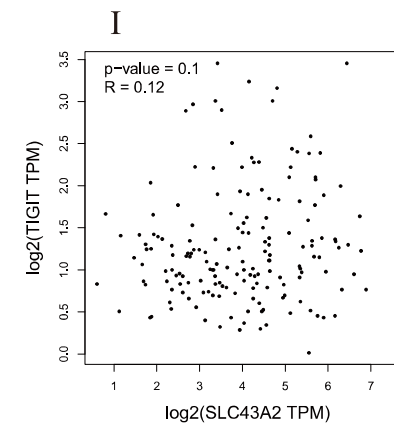

Supplement: Supplementary Figure 1 — Immune infiltration analysis of SLC43A2 genes in AML.SLC43A2 was positively correlated with infiltration score (A), cytotoxic cells (B), dendritic cells (C), exhausted cells (D), and macrophage cells (E), monocyte infiltration score (F) and neutrophil infiltration score (G). [file DataSheet1.zip › Supplementary Figure 3.pdf]

A

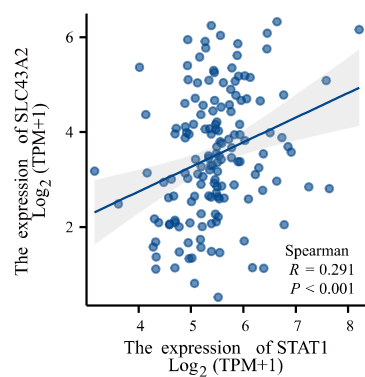

B

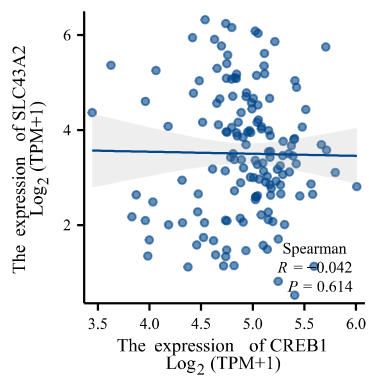

C

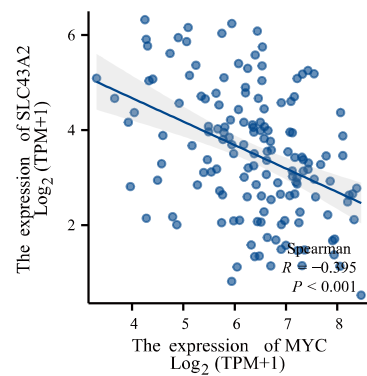

D

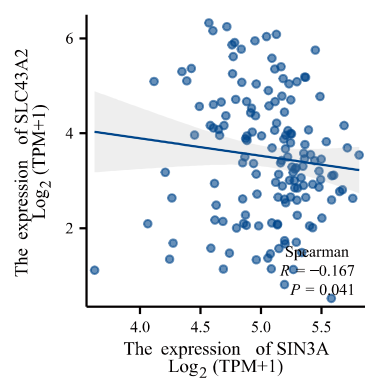

E

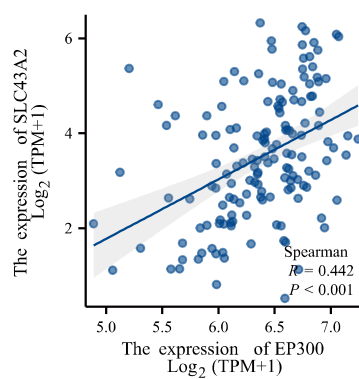

F

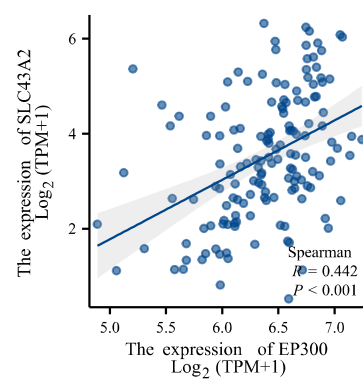

G

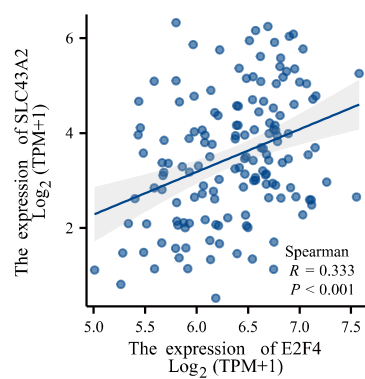

Supplement: Supplementary Figure 1 — Immune infiltration analysis of SLC43A2 genes in AML.SLC43A2 was positively correlated with infiltration score (A), cytotoxic cells (B), dendritic cells (C), exhausted cells (D), and macrophage cells (E), monocyte infiltration score (F) and neutrophil infiltration score (G). [file DataSheet1.zip › Supplementary Figure 4.pdf]
